# The mechanism of bending in co-crystals of caffeine and 4-chloro-3-nitrobenzoic acid

**DOI:** 10.1038/s41467-021-26204-z

**Published:** 2021-10-20

**Authors:** Amy J. Thompson, Jason R. Price, John C. McMurtrie, Jack K. Clegg

**Affiliations:** 1 School of Chemistry and Molecular Biosciences, The University of Queensland, St Lucia, QLD 4072 Australia; 2 ANSTO Melbourne, The Australian Synchrotron, 800 Blackburn Rd, Clayton, VIC 3168 Australia; 3 School of Chemistry and Physics, Faculty of Science and Engineering, Queensland University of Technology (QUT), 2 George Street, Brisbane, QLD 4000 Australia; 4 Centre for Materials Science, Queensland University of Technology (QUT), 2 George Street, Brisbane, QLD 4000 Australia

**Keywords:** Mechanical properties, Crystal engineering, Organic molecules in materials science

**arising from** Somnath Dey et al. *Nature Communications* 10.1038/s41467-019-11657-0 (2019)

In a recent study, Dey et al.^1^ propose a mechanism of elastic bending in co-crystals of caffeine, 4-chloro-3-nitrobenzoic acid and methanol (**1**) in which mechanical interlocking is proposed to allow for the reversible flexibility observed. We have now determined the mechanism to atomic resolution using micro-focused synchrotron radiation^2^, which is different to that previously reported. When subjected to strain the intermolecular distances change and hydrogen-bonded dimers rotate over two orthogonal directions to allow the compression and expansion producing flexibility.

Dey et al.^1^ used an adaptation of the published method^3,4^ with a scan width of 3° and a full 180° rotation to crystallographically investigate **1** at three positions across a bent crystal at room temperature, which produced some striking results. Firstly, the application of strain led to changes in bond lengths. Secondly, the inner length of the crystal appeared to be longer than the centre, while the reported changes in interlocking were not linear. Thirdly, the neutral axis of the crystal was shifted from the centre. This is unexpected from Euler–Bernoulli beam theory^5^. These findings led us to further investigate the responses of this system upon bending.

In our investigation, crystals of **1** were prepared^6^ and confirmed to have the same crystal structure and habit as previously reported (orthorhombic, *Fdd2*). The crystals were face indexed and found to have the orientation provided in Fig. 1, consistent with the predicted morphology arising from BFDH calculations^7^, but different from that previously reported^6^. Within the crystals, molecules of caffeine and 4-chloro-3-nitrobenzoic acid form hydrogen-bonded dimers which stack through π-interactions defined by the *c*-axis. The mean planes of the molecules are perpendicular to either the (110) or ($\bar{1}10$) face.

**Fig. 1:**
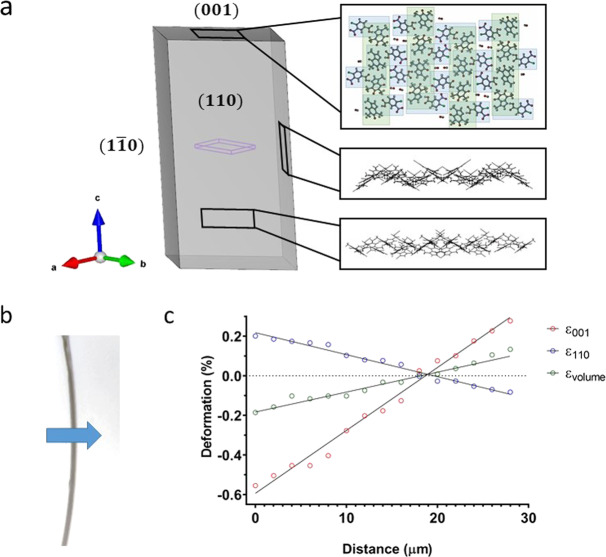
The crystal structure of 1 and the deformation of a bent crystal. **a** Crystal packing of **1** with respect to the face-indexed crystal. The interlocked comb-like structure on the (001) face is emphasised through the green (hydrogen bonding) and blue rectangles. The wireframe view of the (110) and ($\bar{1}10$) faces emphasises the molecules which have their mean planes perpendicular to the face. **b** A bent co-crystal of **1** with thickness of ~38 µm mounted for analysis. The blue arrow indicates the area covered by the mapping experiment and the direction it was performed in. **c** Deformation graph of **1**. Note that ε(110) is equivalent to ε($1\bar{1}0$) due to symmetry. The first dataset containing reflections is defined at 0 µm. For each data set the crystal was rotated through a 30° wedge (15° either side of the centre). The beam was centred off the edges of the crystal, which is ~38 µm thick, at the start and end of the experiment. The centre of the crystal is located at ~ 19 µm. Some data sets beyond 28 µm did not refine satisfactorily due to their position near the edge of the crystal and have been excluded from the analysis.

A crystal of **1** was then bent and subjected to micro-focused synchrotron analysis at 100(2) K^3^. A 30° wedge of data was collected every 2 µm across the apex of the bend using a 10 µm beam. An 80 µm transect covering the entire 40 µm width of the crystal was measured, mapping changes in the unit cell throughout the sample (Fig. 1, all CIFs are available from the CCDC and data analysis is presented in the Supplementary Information). As expected, the *c*-axis, which is coincident with the length of the crystal is compressed in the interior of the bend and expanded on the exterior. The neutral axis is located in the centre of the crystal.

With the change in the *c*-axis of the bent crystal from the inside to the outside, the molecules interacting through π-stacking move further apart. Additionally, the angle between the mean plane of the hydrogen-bonded dimers and the (001) plane increases (Fig. 2). This causes expansion in the *ab*-plane on the inside of the crystal, while the opposite occurs on the outside of the crystal. Equal numbers of molecules have their mean planes perpendicular to the (110) and ($1\bar{1}0$) faces, causing the deformation to occur equally in these directions. Therefore, the molecules both rotate and move apart in response to strain (Fig. 2). There is no change in the hydrogen bond, nor any other bond lengths within the molecules. While similar to the mechanism reported for [Cu(acac)_2_]^3,8^, the changing separation of the π-stacked molecules and the symmetry of **1** distinguish this new mechanism as the molecular rotations in this case are bidirectional.

**Fig. 2:**
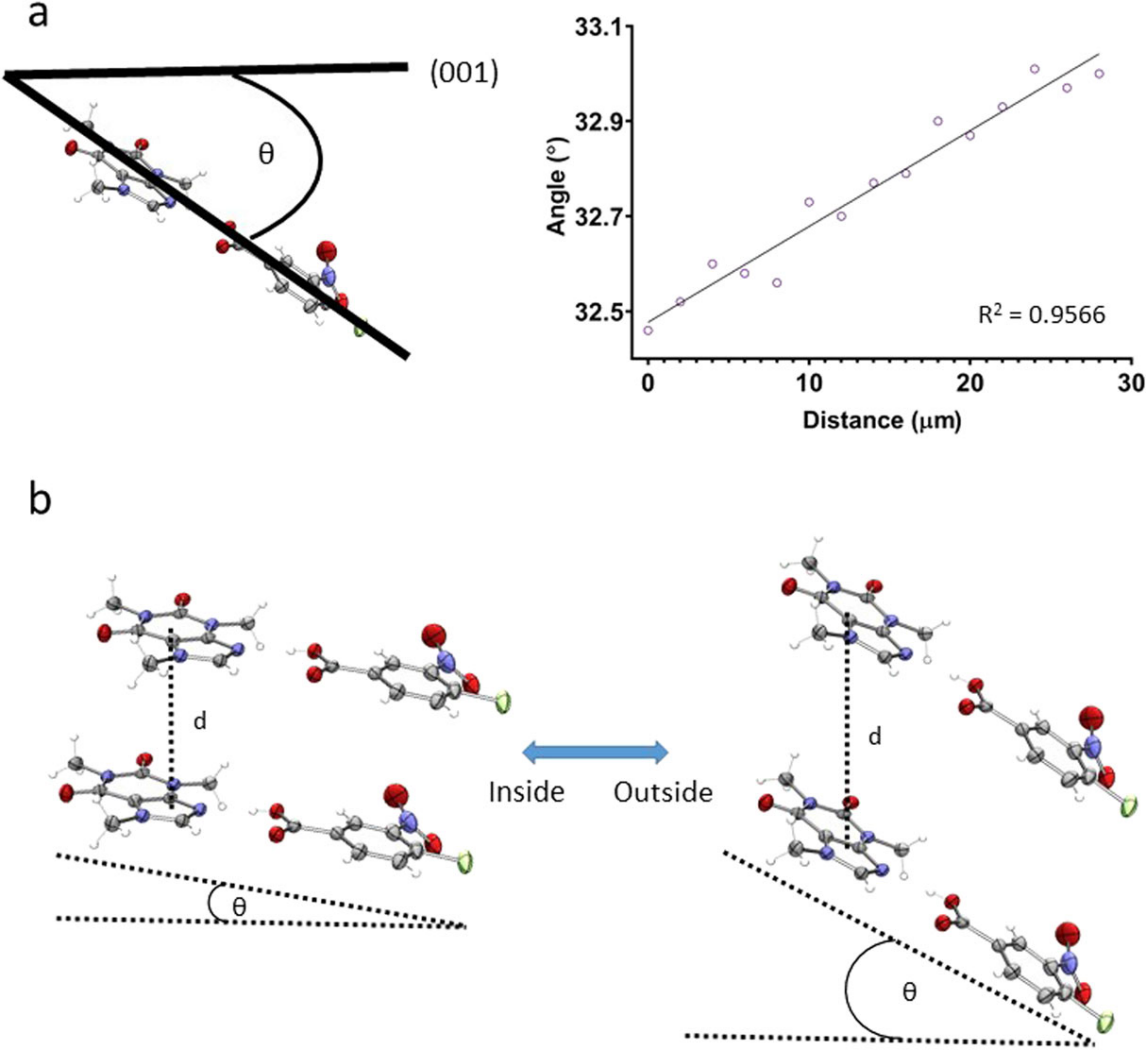
Proposed mechanism of elastic flexibility in co-crystals of 1. **a** The change in angle between the mean plane of the hydrogen-bonded dimer and the (001) plane moving from the inside to the outside of the bend. **b** Rotation compared to the (001) face and movement apart within the π-stack (d) facilitates the required cell deformation. Δd = 0.04 Å, Δθ = 0.54°. Disorder in the nitro-group is removed for clarity.

Dey et al. attributed changes in the angle of 1D caffeine tapes running along the [103] and [$10\bar{3}$] directions as an indication of “interlocking” and the mechanism of flexibility^1^. While our data shows the same angle changes, it arises due to cell deformation (see Supplementary Information). The changes in the angle between these tapes does not account for the observed deformation in the [010] direction. The molecular movement illustrated in Fig. 2 accounts for all observed deformations in the crystal and describes the molecular mechanism of elastic flexibility in these co-crystals. The variation in the extent of “interlocking” is not the mechanism of flexibility. While Dey et al. estimate that their sample was subjected to higher elastic strain (3%) than our crystals (0.6%), the mechanism of elastic behaviour must be the same within the linear region of elastic response. The temperature differences between the two measurements are also unlikely to change the observed mechanism of elastic behaviour^7^, as no phase transition was observed within this temperature range.

In summary, the mechanism of elasticity in co-crystals of caffeine, 4-chloro-3-nitrobenzoic acid and methanol involves a combination of molecular rotation and movement to form the necessary compression and expansion upon bending. Molecular interlocking may prevent slippage between layers of molecules which would lead to plastic deformation. These results provide new insights into the mechanisms of bending in elastically flexible crystals^9^.

## Supplementary information

Supplementary Information

## Data Availability

The crystallographic information files for the structures reported in this study have been deposited at the Cambridge Crystallographic Data Centre (CCDC), under deposition numbers 2012127-2012142. These data can be obtained free of charge from the Cambridge Crystallographic Data Centre via www.ccdc.cam.ac.uk/data_request/cif.
