# Supplementary material for: The mechanism of bending in co-crystals of caffeine and 4-chloro-3-nitrobenzoic acid
Source: Nat Commun. 2021 Oct 20;12:5983. doi: 10.1038/s41467-021-26204-z (PMC8528856; doi:10.1038/s41467-021-26204-z)
Supplement: Supplementary file 1 — Supplementary Information [file 41467_2021_26204_MOESM1_ESM.pdf]

Supplementary Information for The Mechanism of Bending in Co-Crystals of Caffeine and 4-Chloro-3-Nitrobenzoic Acid

Matters arising from: Dey, S.; Das, S.; Bhunia, S.; Chowdhury, R.; Mondal, A.; Bhattacharya, B.; Devarapalli, R.; Yasuda, N.; Moriwaki, T.; Mandal, K.; Mukherjee, G. D.; Reddy, C. M., Mechanically interlocked architecture aids an ultra-stiff and ultra-hard elastically bendable cocrystal. *Nat Commun.* **10** (1), 3711 (2019).

Amy J. Thompson,[1] Jason R. Price,[2] John C. McMurtrie*[3, 4] and Jack K. Clegg*[1]

[1]School of Chemistry and Molecular Biosciences, The University of Queensland, St Lucia, Qld 4072, Australia. j.clegg@uq.edu.au

[2]ANSTO Melbourne, The Australian Synchrotron, 800 Blackburn Rd, Clayton, Vic 3168, Australia.

[3]School of Chemistry and Physics, Faculty of Science and Engineering, Queensland University of Technology (QUT), 2 George Street, Brisbane, QLD 4000, Australia. j.mcmurtrie@qut.edu.au

[4]Centre for Materials Science, Queensland University of Technology (QUT), 2 George Street, Brisbane, QLD 4000, Australia.

Experimental

Caffeine and 4-chloro-3-nitrobenzoic acid, which were obtained from commercial sources, were dissolved in hot methanol in a 1:1 ratio according to previous procedures[1]. Clear, acicular crystals were grown from the slow evaporation of this solution.

Face Indexing

Single crystal data was collected using the XtaLAB Synergy Dualflex configured to use graphite monochromated Mo-Kα radiation from a sealed tube (0.71073 Å) with ω and ψ scans at 100(2) K. Data collection, integration, reduction and face indexing (Supplementary Figure 1) were performed using the software package CrysAlis Pro[2]. As the crystal was very small (more suited to synchrotron radiation) and experienced significant wobbling during data collection it was not refined to completion, although the indexation and structure solutions were consistent with other data.

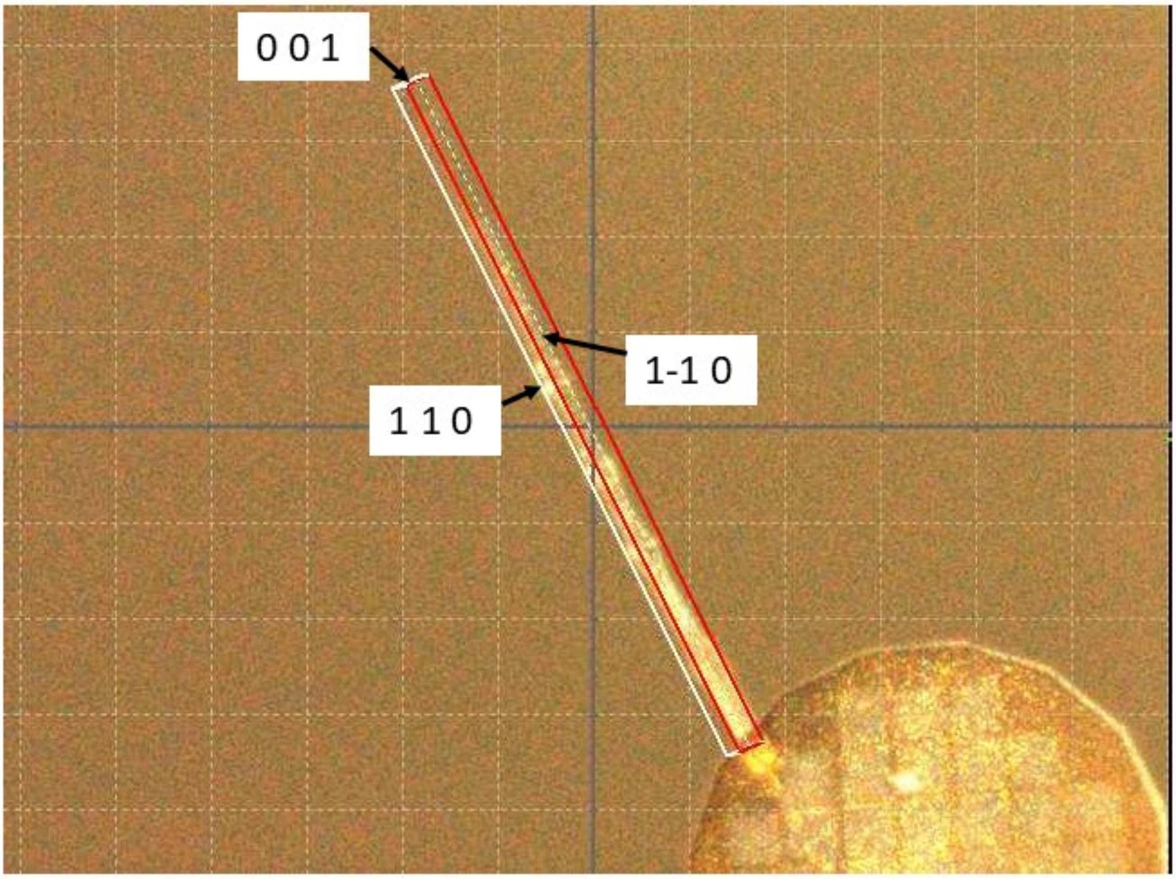

Supplementary Figure 1 - Face indexing of the co-crystal

Experimental for Synchrotron Measurements

Micro-focus SCXRD for mapping experiments were performed at the Australian Synchrotron MX2 beamline[3]. All of the measurements were performed at 100(2) K using the wavelength $\lambda = 0.7108$ Å. A full data collection was performed with a beam cross-section (full-width at half-maximum (FWHM)) of 22 x 12 µm. Mapping studies were performed using a micro-collimator that produced a beam cross-section of 10 x 11.25 µm (FWHM). Data acquisition was performed using AS QEGUI[4]. Data for three separate bent crystals were collected in 30° wedges to minimise interference from the shoulders of the crystal, moving 2 µm between collections. Data integration and reduction was performed using the XDS package[5]. The unbent structure (collected as a full 360° ϕ scan) was solved with ShelXT[6] and refined with ShelXL[7] using the Olex2 graphical interface[8]. Data from the mapping experiments were refined against the reference solution using ShelXL[7]. All structures were refined isotropically and disorder left unmodelled to maintain reasonable data to parameter ratios. Full CIF files for the primary mapping data set are supplied. The isotropic refinement and limited number of

diffraction images collected for the mapping studies results in a number of CheckCIF Level A and B alerts. CCDC numbers 2012127-2012142.

Reference Structure

The crystal structure of the co-crystal measured using synchrotron radiation is given in Supplementary Figure 2, with one of each molecule in the asymmetric unit. As the molecule of methanol is heavily disordered, SQUEEZE[9-11] was implemented. Disorder was also present in the nitro group. Crystal Data for $C_{15}H_{14}ClN_5O_6$ (M =395.76 g/mol): orthorhombic, space group Fdd2 (no. 43), a = 32.795(7) Å, b = 55.467(11) Å, c = 3.9660(8) Å, V = 7214(3) Å^3, Z = 16, T = 99.98(2) K, μ(MoKα) = 0.256 mm^{-1}, $Dcalc$ = 1.457 g/cm^3, 30980 reflections measured (2.886° ≤ 2Θ ≤ 58.256°), 4868 unique (R_{int} = 0.0772, R_{sigma} = 0.0416) which were used in all calculations. The final R_1 was 0.0415 (I > 2σ(I)) and wR_2 was 0.1146 (all data).

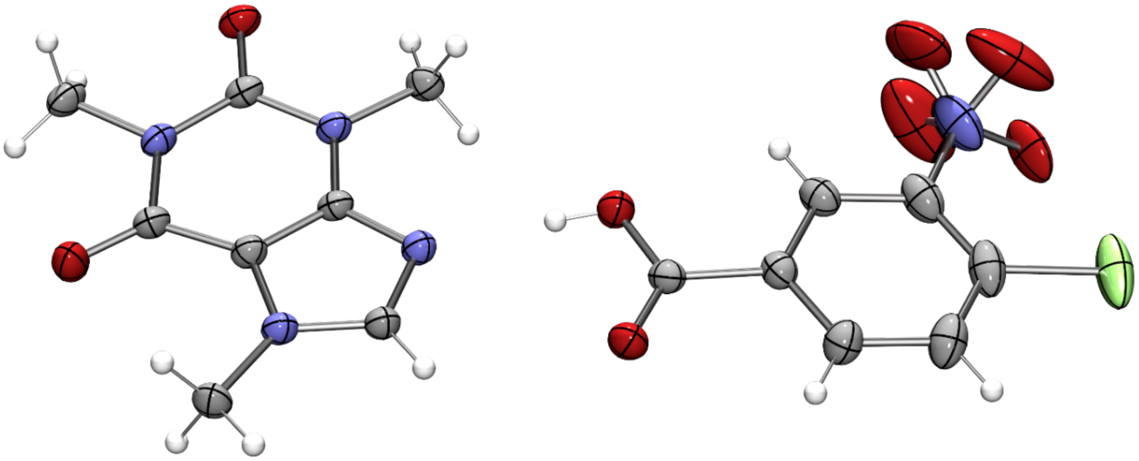

Supplementary Figure 2 - ORTEP[12] representation of the co-crystal. Atoms are represented as 50% probability thermal ellipsoids. Note the disorder of the NO_2 group.

In order to maintain reasonable data:parameter ratios for the mapping data set, the reference structure used was refined isotropically with no modelled disorder. SQUEEZE was not used for the reference, and instead the methanol was modelled as a single entity.

Mapping Data

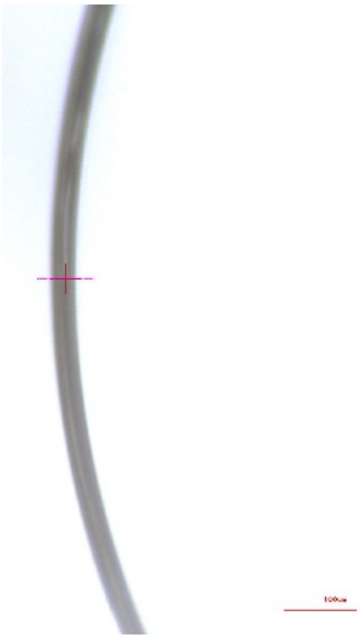

Supplementary Figure 3 - Bent crystal mounted for a mapping experiment. Note the purple bar which indicates the range of data collection.

The crystal in Supplementary Figure 3 was bent (strain estimated to be 0.6%), mounted and analysed to produce the deformation graph in Supplementary Figure 4. As the system is orthorhombic, the directions between the (110)/(-1-10) and (1-10)/(-110) pairs of faces experience identical percentage deformation. The data was fitted with a series of linear lines constrained to share their crossing point, which resulted in an intersection at 0% deformation within the 95% confidence interval. R^2 values are given in Supplementary Table 1 - Equations and statistics for the lines of best fit in Supplementary Figure 4. All trends were found to be statistically significant via an F-Test with P-values all <0.0001.

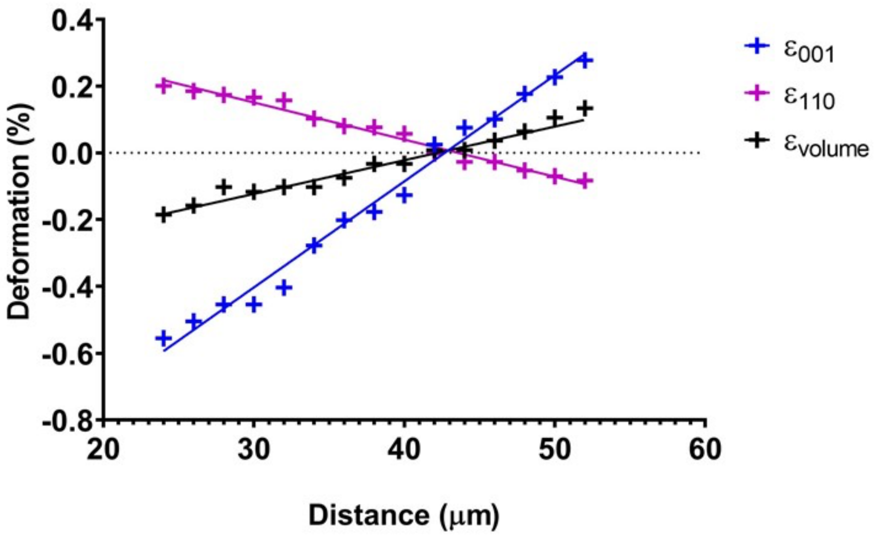

Supplementary Figure 4 - Deformation and lines of best fit for the co-crystal measured from the inside to the outside of the bend

	ε_{001}	ε_{110}	ε_{volume}
Equation	$y = 0.0318x - 1.356$	$y = -0.0109x + 0.4806$	$y = 0.0102x - 0.4265$
R^2 value	0.9865	0.9779	0.9652

Supplementary Table 1 - Equations and statistics for the lines of best fit in Supplementary Figure 4

These trends were confirmed by testing a further two crystals (Supplementary Figure 5 and Supplementary Figure 6). While the crystals were of a lower quality, the same trends can be observed demonstrating the reproducibility of the results.

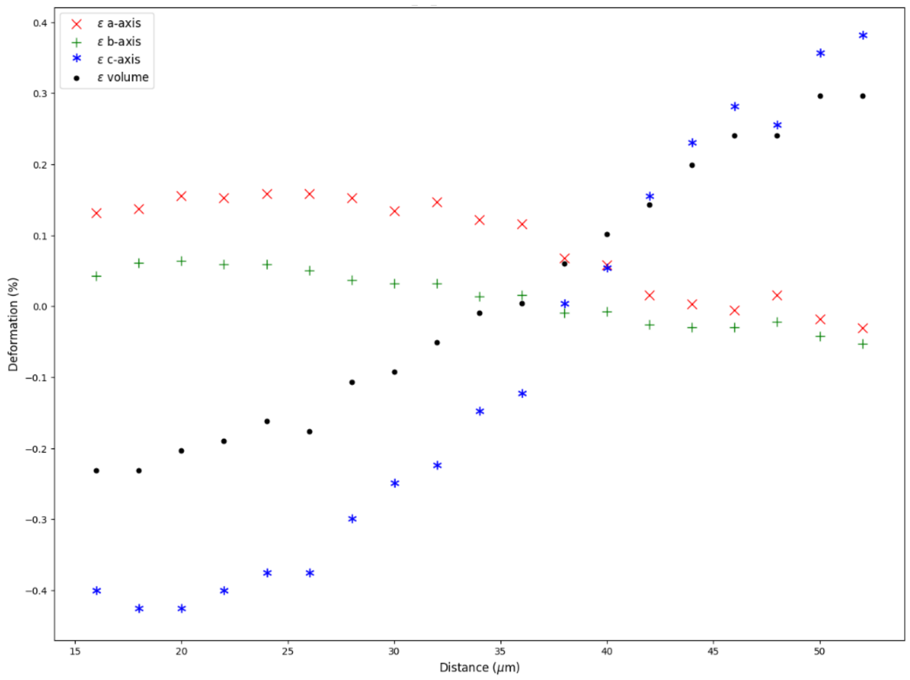

Supplementary Figure 5 - Raw mapping data from a second crystal

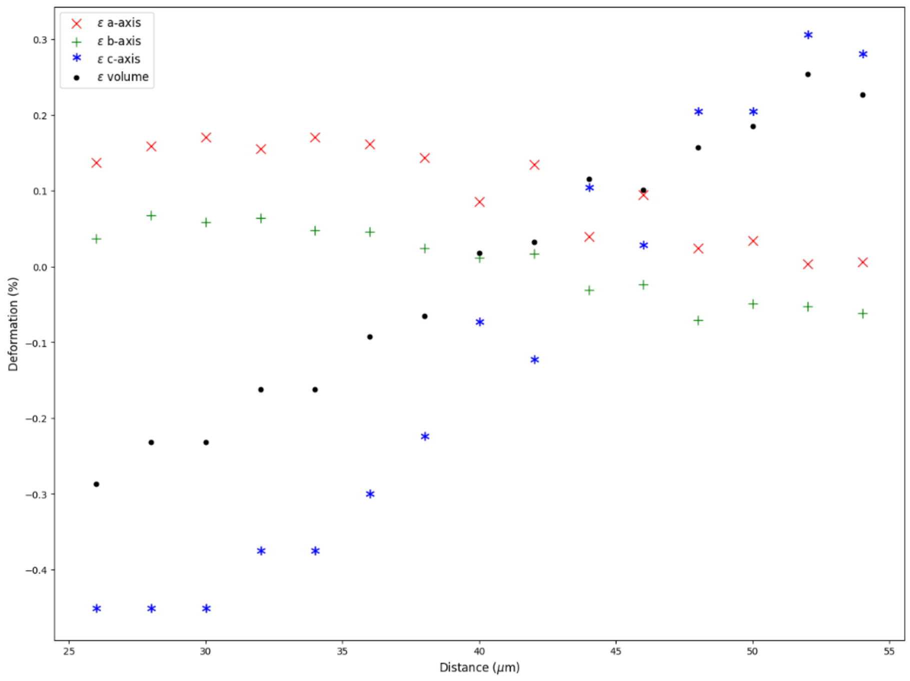

Supplementary Figure 6 - Raw mapping data from a third crystal

Previous reports[13] demonstrated that the neutral position, or point of no deviation from the unbent crystal occurred on the inside of the sides, and the outside at the apex of the bend (see previously reported Fig. 3[13] for graphical depiction). The left and right sides of a mounted crystal were also mapped (Supplementary Figure 7). It should be noted that due to limitations

in the experimental set up, the region mapped was not exactly perpendicular to the width of the crystal, however the trends in Supplementary Figure 8 do show quite convincingly that the neutral position is likely in the centre of the crystal (position at ~40 μm). Further structures on the outside of the right hand side were not able to be solved due to poor diffraction quality. All trends were found to be statistically significant via and F-Test with P-Values all <0.0001, and the equations and R^2 values can be found in Supplementary Table 2.

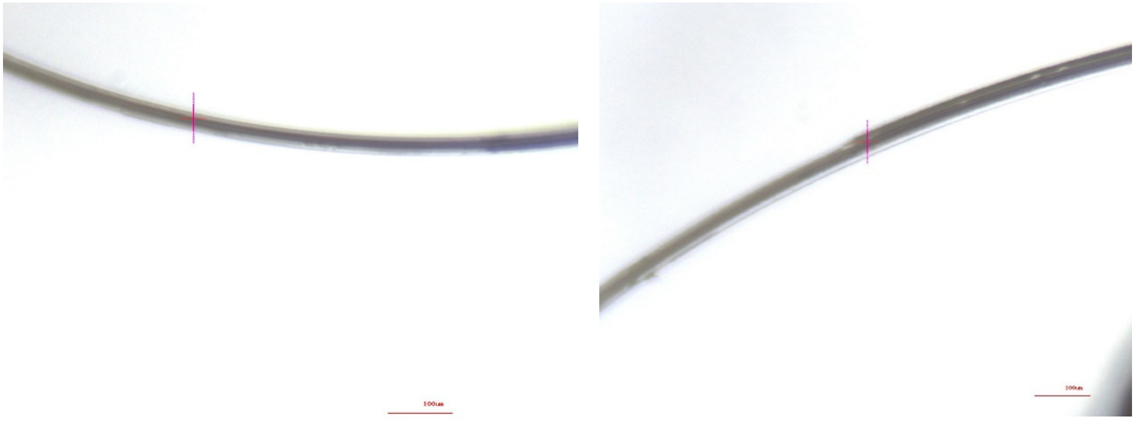

Supplementary Figure 7 - Left and right sides of a bent crystal for mapping both mapped from the inside to the outside.

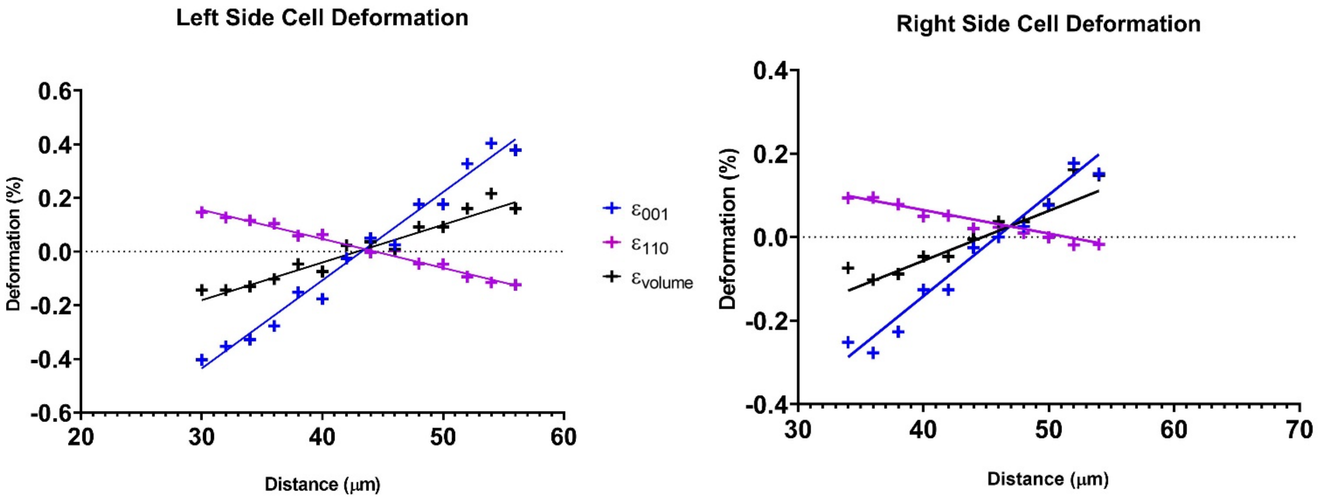

Supplementary Figure 8 - Deformation on the left and right hand side of the crystal from the inside to the outside.

**Left**	ε_{001}	ε_{110}	ε_{volume}
Equation	$y = 0.0329x - 1.422$	$y = -0.0108x + 0.4831$	$y = 0.0141x - 0.6013$

R^2 value	0.9779	0.9831	0.9406
**Right**	ε_{001}	ε_{110}	ε_{volume}
Equation	$y = 0.0242x - 1.112$	$y = -0.0056x + 0.2901$	$y = 0.0120x - 0.5347$
R^2 value	0.9599	0.9258	0.8698

Supplementary Table 2 - Equations and statistics for the lines of best fit in Supplementary Figure 8.

Atom Labelling Scheme For Mechanism Analysis

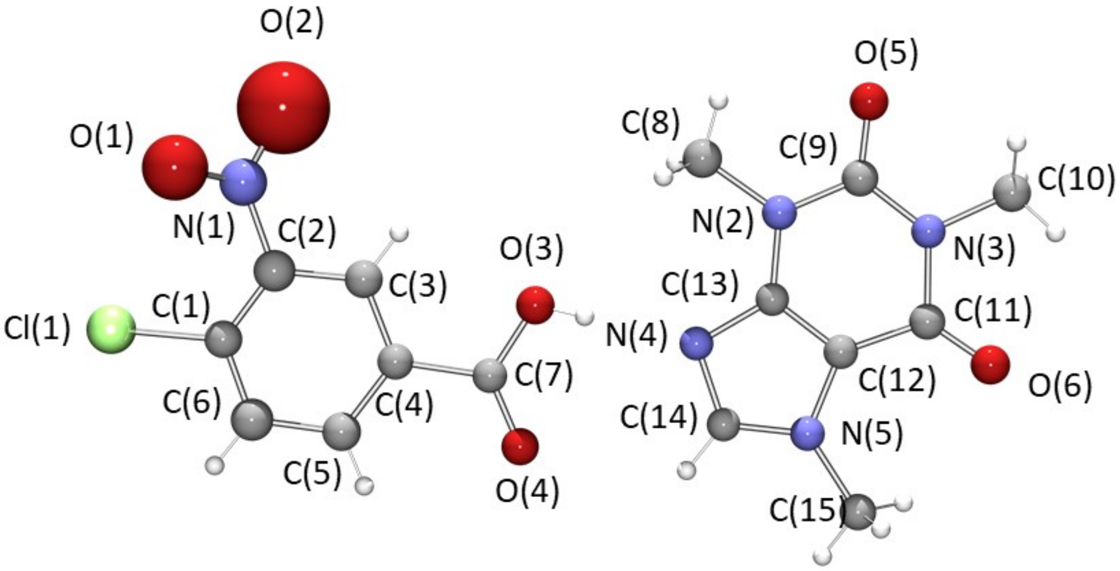

Supplementary Figure 9 - Atom labelling scheme

Additional Mechanistic Insights

The rotation mechanism from the parent paper is further supported by the following additional graphs. Firstly, it was confirmed that the distance between two hydrogen-bonding molecules was consistent. This was done by measuring O(3) – N(4) and O(4) – C(14). As Supplementary Figure 10 shows, there were no statistically significant correlations confirmed through an F-Test.

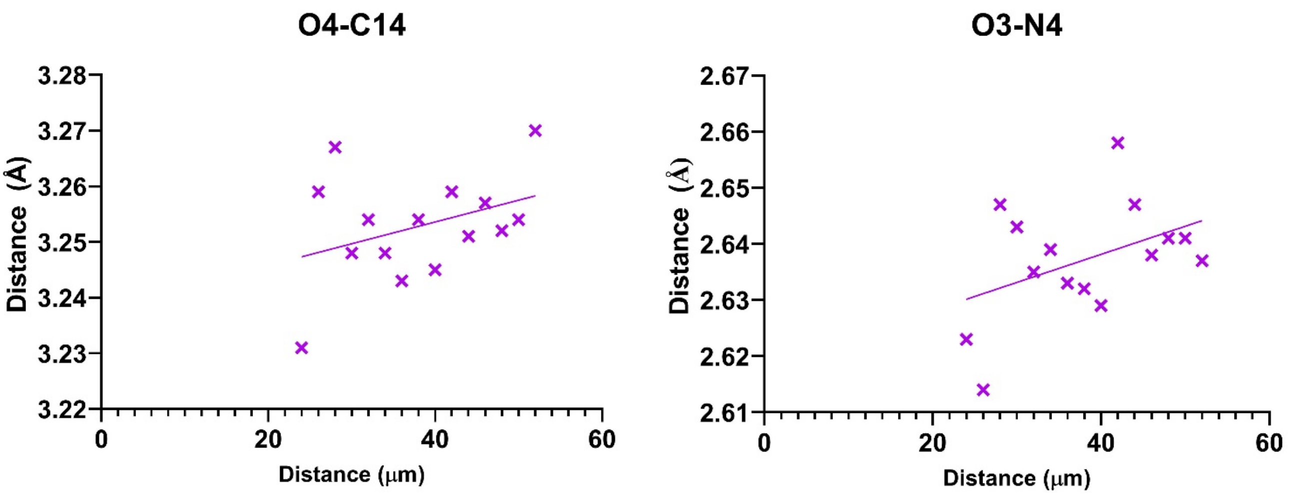

Supplementary Figure 10 - Measured distances between a hydrogen-bonded dimer

Comparisons to Previous Mechanism

Various changes in bond lengths were shown in previous reports[13]. These quoted changes were investigated as shown in Supplementary Figure 11 (note the atom labelling scheme in Supplementary Figure 9). Statistical analysis was applied to each graph, with an F-test revealing that the only change with a significant correlation was the C(4) – C(7) bond length. However, the R^2 value was only 0.6484, which is significantly lower than the R^2 values quoted from this study. Additionally, based on the graphical mechanism in previous reports (see Fig 5.[13]), it is expected that there would be a significant trend in the distance between Cl(1) and C(15). This is due to the depicted expansion/contraction of the interlocked groups. These distances were also measured, although to a poor R^2 value of 0.3259 (Supplementary Figure 12).

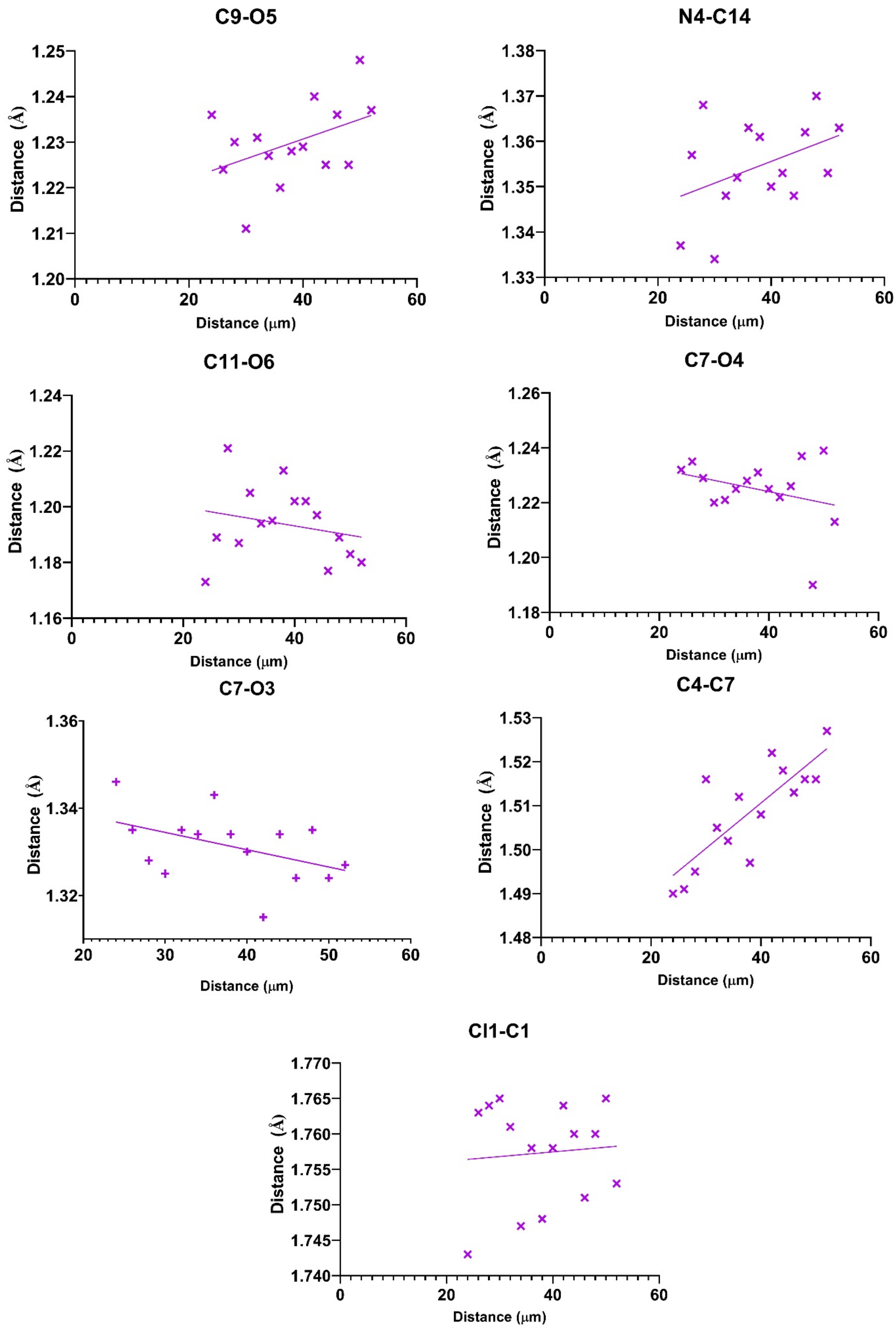

Supplementary Figure 11 - Measurement of bond-lengths based on previous findings

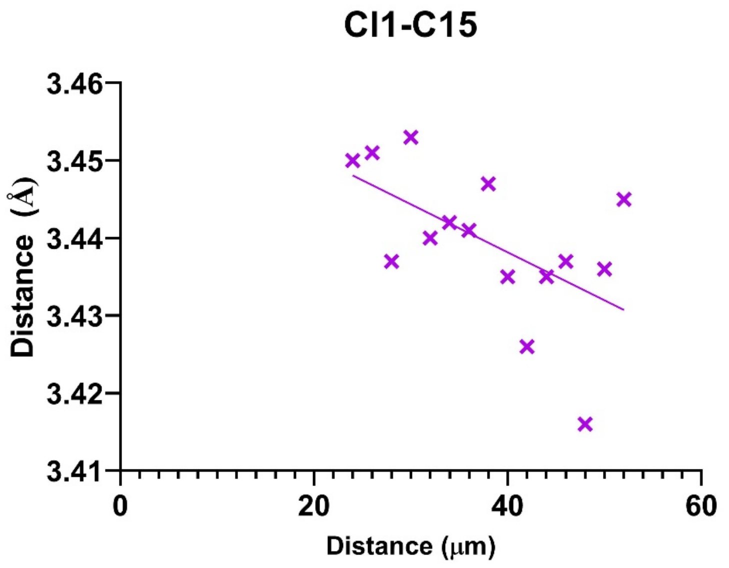

Supplementary Figure 12 - Measurement of intermolecular distances between Cl(1) and C(15)

A further trend was reported between the angle of 1D caffeine tapes along the [103] and [10-3] directions[13]. While our data does confirm this (Supplementary Figure 13), it is only an artefact of the cell deformations. This is shown in Supplementary Figure 14, which details how these angles can be calculated from the a- and c-axes. The perceived rotation along the [103] and [10-3] directions would result in no deformation to the b-axis, although the deformation graph in Supplementary Figure 4 shows a change in the length of this axis across the bend. Therefore, the flexure of the 1D caffeine tapes along the [103] and [10-3] directions is not a suitable mechanism to describe the elastic bending in this co-crystal, whereas the mechanism reported in the parent paper explains the deformation of all three cell axes.

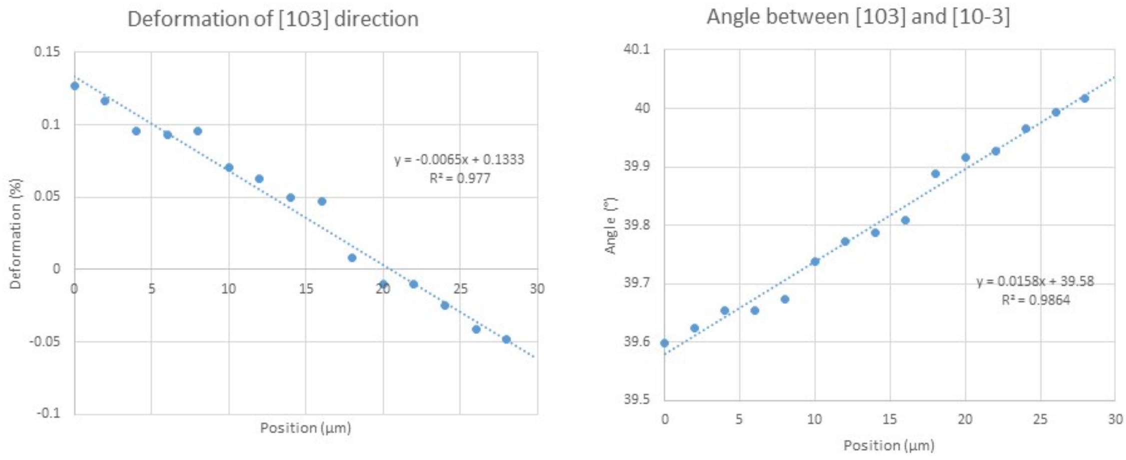

Supplementary Figure 13 – Deformation along the [103] direction (equivalent by symmetry to the [10-3] direction), and the corresponding angle change between 1D caffeine tapes which lie along these directions

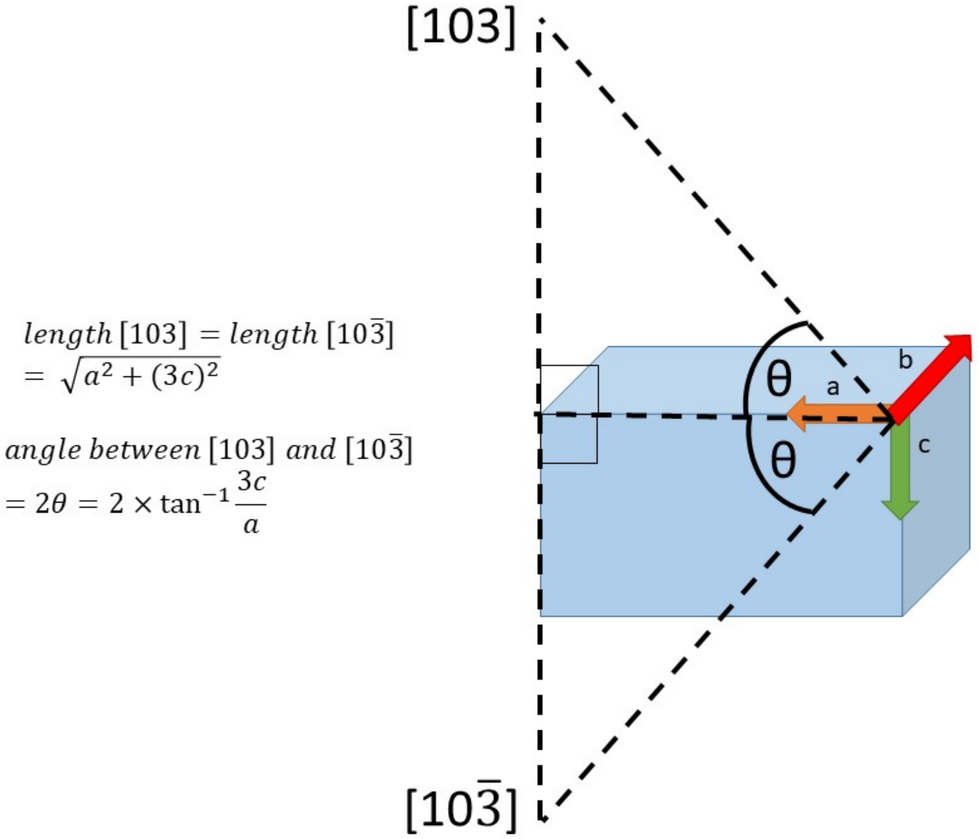

Supplementary Figure 14 – Deformation along [103] and [10-3] directions has no b-component, producing no change in the length of the b-axis. As such, the changing angle between [103] and [10-3] is an artefact of the changing a- and c-axis

Supplementary References

1 Ghosh, S. & Reddy, C. M. Elastic and bendable caffeine cocrystals: implications for the design of flexible organic materials. *Angew. Chem., Int. Ed.*$\mathbf{51}$, 10319-10323, (2012).

2 CrysAlis PRO (Agilent Technologies Ltd, Yarnton, Oxfordshire, England 2014).

3 Aragao, D. *et al.* MX2: a high-flux undulator microfocus beamline serving both the chemical and macromolecular crystallography communities at the Australian Synchrotron. *J Synchrotron Radiat* **25**, 885-891, (2018).

4 AS QEGui (https://qtepics.github.io/, 2019).

5 Kabsch, W. Automatic processing of rotation diffraction data from crystals of initially unknown symmetry and cell constants. *J. Appl. Cryst.* **26**, 795-800 (1993).

6 Sheldrick, G. M. SHELXT - integrated space-group and crystal-structure determination. *Acta Cryst.* **A71**, 3-8, (2015).

7 Sheldrick, G. M. Crystal structure refinement with SHELXL. *Acta Cryst.* **C71**, 3-8, (2015).

8 Dolomanov, O. V., Bourhis, L. J., Gildea, R. J., Howard, J. A. K. & Puschmann, H. OLEX2: a complete structure solution, refinement and analysis program. *J. Appl. Cryst.* **42**, 339-341, (2009).

9 van der Sluis, P. & Spek, A. L. BYPASS: an effective method for the refinement of crystal structures containing disordered solvent regions. *Acta Cryst.* **46**, 194-201, (1990).
10 Spek, A. PLATON SQUEEZE: a tool for the calculation of the disordered solvent contribution to the calculated structure factors. *Acta Cryst.* **C71**, 9-18, d (2015).
11 Spek, A. PLATON: A Multipurpose Crystallographic Tool (Utrecht University, Utrecht, The Netherlands, 2008).
12 Farrugia, L. ORTEP. *J. Appl. Cryst.* **30**, 565 (1997).
13 Dey, S. *et al.* Mechanically interlocked architecture aids an ultra-stiff and ultra-hard elastically bendable cocrystal. *Nat Commun* **10**, 3711, (2019).
